# Association of crumbs homolog-2 with mTORC1 in developing podocyte

**DOI:** 10.1371/journal.pone.0202400

**Published:** 2018-08-20

**Authors:** Sho Hamano, Yukino Nishibori, Ichiro Hada, Naoaki Mikami, Noriko Ito-Nitta, Daisuke Fukuhara, Akihiko Kudo, Zhijie Xiao, Masatoshi Nukui, Jaakko Patrakka, Karl Tryggvason, Kunimasa Yan

**Affiliations:** 1 Department of Pediatrics, Kyorin University School of Medicine, Mitaka, Tokyo, Japan; 2 Department of Anatomy, Department of Pediatrics, Kyorin University School of Medicine, Mitaka, Tokyo, Japan; 3 Division of Matrix Biology, Department of Medical Biochemistry and Biophysics, Karolinska Institute, Stockholm, Sweden; 4 Department of Laboratory Medicine, KI/AZ Integrated CardioMetabolic Center, Karolinska Institute, Karolinska University Hospital Huddinge, Huddinge, Sweden; University of PECS Medical School, HUNGARY

## Abstract

The evidence that gene mutations in the polarity determinant Crumbs homologs-2 (CRB2) cause congenital nephrotic syndrome suggests the functional importance of this gene product in podocyte development. Because another isoform, CRB3, was reported to repress the mechanistic/mammalian target of the rapamycin complex 1 (mTORC1) pathway, we examined the role of CRB2 function in developing podocytes in relation to mTORC1. In HEK-293 and MDCK cells constitutively expressing CRB2, we found that the protein localized to the apicolateral side of the cell plasma membrane and that this plasma membrane assembly required *N*-glycosylation. Confocal microscopy of the neonate mouse kidney revealed that both the tyrosine-phosphorylated form and non-phosphorylated form of CRB2 commence at the S-shaped body stage at the apicolateral side of podocyte precursor cells and move to foot processes in a capillary tuft pattern. The pattern of phosphorylated mTOR in developing podocytes was similar to that of CRB2 tyrosine phosphorylation. Additionally, the lack of a tyrosine phosphorylation site on CRB2 led to the reduced sensitivity of mTORC1 activation in response to energy starvation. CRB2 may play an important role in the mechanistic pathway of developing podocytes through tyrosine phosphorylation by associating with mTORC1 activation.

## Introduction

Mature kidney glomeruli possess a specific ultrafiltration barrier that is composed of fenestrated endothelial cells, a thick basement membrane, and visceral epithelial cells termed podocytes. A podocyte is a terminally differentiated post-mitotic cell that exhibits a specific shape similar to that of a neuronal cell, branching into primary and secondary foot processes from the cell body [1]. A specific and essential structure termed the slit diaphragm (SD) is present in the spaces between the interdigitating secondary foot processes that branch in opposite directions. The SD is the outermost filtration barrier covering the glomerulus and limits plasma proteins based on their size. Pathologically, the apparent loss or dislocation of the SD in diseased glomeruli is observed in nephrotic syndrome, a major cause of podocytopathies with massive proteinuria.

The developmental process of podocytes is orchestrated by a very complex pathway [2]. First, podocyte precursor cells form a line along the immature glomerular basement membrane on their basal side, whereas their apical sides are connected to each other through intercellular junctions. During the capillary loop stage, immature podocytes begin to interdigitate with each other at primary processes on the basal side, followed by the formation of secondary processes (foot processes). During this drastic change in cell shape, the apical intercellular junction moves from the cell body of the podocyte precursor cell to the basolateral side of the foot processes of a mature podocyte, eventually transforming into the SD. Achieving this basolateral interdigitation of foot processes requires the dramatic growth and extension of the basal portions of immature podocytes. However, the precise mechanistic and metabolic pathways for this change in cell shape and size and for the transition of the intercellular junction are poorly understood.

Crumbs homolog (CRB) proteins function as a polarity determinant of apical and basolateral membrane domains in epithelial cells and are evolutionarily conserved from flies to humans [3–5]. CRBs participate in intracellular signaling pathways to maintain epithelial homeostasis [6–9]. Three homologs (CRB1, CRB2, and CRB3) are found in mammalian epithelial cells, all of which possess a conserved intracellular domain comprising FERM (band 4.1/ezrin/radixin/moesin) and PDZ (PSD-95/Discs large/ZO-1) motifs and a tyrosine phosphorylation site [10, 11]. A previous study showed that phosphorylation of the CRB2 (ENSMUSG00000035403) cytoplasmic domain disrupts its binding to moesin and allows for it to instead form a complex with Pals1 [12]. CRB1 and CRB2 contain a large extracellular domain with epidermal growth factor-like repeats and laminin globular domains, whereas CRB3 lacks the extracellular domain [11]. Among CRB proteins, CRB2 is expressed in podocytes. Gene knockdown of zebrafish CRB2 leads to glomerular foot process effacement and loss of the SD structure [13]. Moreover, CRB2 mutation was reported to be a novel cause of steroid-resistant nephrotic syndrome [14, 15]. Interestingly, a previous study demonstrated that CRB3 is a suppressor of mechanistic/mammalian target of rapamycin complex 1 (mTORC1) [16]. The activation of mTORC1 plays a fundamental role in protein synthesis and cell growth; however, it remains unknown how the mTORC1 pathway is involved in the developing podocyte [17–19]. Therefore, the aim of this study was to determine whether CRB2 is involved in the mTORC1 pathway in developing podocytes.

## Materials and methods

### Animal experiments

All animal experiments were carried out at the Laboratory Animals Center of Kyorin University School of Medicine and approved by the Committee on Research Animal Care of Kyorin University (permit number: 71–3). 3-day-old newborn and adult (4-week-old) C57BL/6 male mice (CLEA Japan, Tokyo, Japan) were euthanized through cardiac puncture under isoflurane inhalation and intraperitoneal injection with pentobarbital. An incision was made at midline of the abdomen and kidneys were removed and processed for immunohistochemistry, immunofluorescence and confocal microscopy. Pure glomeruli were isolated using the magnetic beads perfusion method, as described previously [20].

### Antibodies

An antibody against the human CRB2 protein (CRB2-Ab) was generated by purifying recombinant protein with an affinity tag and immunizing NZW rabbits (National Veterinary Institute, Uppsala, Sweden) using a standard protocol. Briefly, residues 597 to 943 of the extracellular domain of human CRB2 were cloned into the pET-28a (+) expression vector (Novagen, Madison, WI, USA). The his-tagged CRB2 recombinant protein was solubilized from inclusion bodies in 8 M urea and purified using sequential TALON Superflow^TM^ (Clontech, Mountain View, CA) and Sephadex S-75 gel filtration (GE Healthcare, Uppsala, Sweden). A polyclonal antibody specific for phospho-CRB2 (phospho-CRB2-Ab) was generated against a synthetic peptide corresponding to the tyrosine phosphorylation site at residues 1249–1260 (RRQSEGTpYSPSQ) of mouse CRB2. This peptide was bound to Keyhole Limpet Hemocyanin and administered with Freund’s complete adjuvant. A rabbit was immunized with 0.4 mg of this peptide, boosted 5 times, and then euthanized and bled at 45 days after the first immunization. The immunoglobulin G fraction was purified using an Activated Thiol Sepharose 4B peptide column (GE Healthcare). The mouse monoclonal antibody against human podocalyxin was previously described [21]. The following antibodies were purchased from the suppliers, as indicated: anti-phospho-S6 ribosomal protein (RPS6) (Ser235/236) rabbit monoclonal antibody, anti-RPS6 rabbit monoclonal (5G10) antibody, anti-phospho-mTOR (Ser2448, 49F9, immunohistochemistry specific) rabbit monoclonal antibody, anti-phospho-mTOR (Ser2448) rabbit polyclonal antibody and anti-mTOR rabbit polyclonal antibody (Cell Signaling, Danvers, MA, USA); anti-Wilms’ tumor1 (WT1) mouse monoclonal antibody (6FH2, Dako, Carpinteria, CA, USA); anti-glucose-regulated protein 78 mouse monoclonal antibody: KDEL (Stressgen Biotechnologies, Victoria, Canada); anti-ZO1/TJP1 mouse monoclonal antibody (ZO1-1A12) (Thermo Fisher Scientific); anti-synaptopodin mouse monoclonal antibody (PROGEN, Heidelberg, Germany); anti-CD31/DIA-310 rat monoclonal antibody (OPTISTAIN, Miami, FL, USA); anti-β-actin mouse monoclonal and anti-FLAG M2 mouse monoclonal antibodies (Sigma, St. Louis, MO, USA); HRP-labeled goat anti-rabbit and anti-mouse immunoglobulins (Dako); and Alexa Fluor 488-conjugated goat anti-rabbit IgG, Cy3-conjugated goat anti-mouse IgG (Molecular Probes, Eugene, OR, USA).

### Plasmid construction

A full-length cDNA clone of mouse wild-type CRB2 was obtained from a mouse kidney cDNA library, excised using EcoRI and subcloned into the EcoRI site of the mammalian expression vector pcDNA3.1/Zeo(-) (Invitrogen, Carlsbad, CA, USA). The sequence was confirmed using a BigDye Terminator cycle sequencing kit (Applied Biosystems, Foster City, CA). Mutation of the CRB2 phosphorylation site (Y1255F) was performed using the mouse wild-type CRB2 full-length cDNA expression vector as a template and a KOD-Plus-Mutagenesis Kit according to the manufacturer’s protocol (TOYOBO, Osaka, Japan). The full-length cDNA encoding mouse CRB2 was subcloned into a HindIII/XbaI-cleaved mammalian expression vector p3XFLAG-CMV-14 (Sigma, St. Louis, MO) using an In-Fusion Cloning Kit according to the manufacturer’s manual (TaKaRa, Shiga, Japan).

### Cell lines and cell culture

A conditionally immortalized mouse podocyte cell line was cultured and maintained as previously described ([22, 23] kindly donated from Dr. Katsuhiko Asanuma). Human Embryonic Kidney 293 (293) cells were grown in Dulbecco’s modified Eagle’s medium (D-MEM, Life Technologies, Gaithersburg, MD, USA) supplemented with 10% fetal calf serum and 100 U/mL penicillin in a 37°C incubator with 5% CO_2_. Madin-Darby Canine Kidney (MDCK) cells were grown in Roswell Park Memorial Institute-1640 medium (RPMI-1640, Life Technologies) supplemented with 10% fetal calf serum and 100 U/mL penicillin. For the generation of stably expressing mouse CRB2 cell lines (293-CRB2, 293-CRB2-phospho-mutant, 293-Flag-CRB2, MDCK-CRB2, MDCK-CRB2-phospho-mutant), HEK-293 cells and MDCK cells were transfected with 10 μg of each CRB2 plasmid DNA using FuGene6^TM^ (Roche Diagnostics, Mannheim, Germany) according to the manufacturer’s protocol. The cells were selected and cultured in medium containing 500 μg/mL G418 (Sigma-Aldrich) or 300 μg/mL Zeocin (Thermo Fisher Scientific).

293-CRB2 cells were cultured in each growth medium before tunicamycin treatment. The cells were washed once with phosphate-buffered saline (PBS) and cultivated for 20 h in fresh culture medium in the absence or presence of tunicamycin (5 μg/ml). Protein samples were subjected to immunoblotting of CRB2. 293-CRB2 cells were cultured on a cover slip and treated with tunicamycin as above. The cells were then subjected to surface immunostainig and intracellular staining as described below.

### Reverse transcription polymerase chain reaction (RT-PCR)

Total RNA was extracted from an immortalized mouse podocyte cell line cultured at 37°C and isolated mouse glomeruli using Isogen (Wako Life Science Reagents, Osaka, Japan) according to the manufacturer’s instructions. The reverse transcription polymerase chain reaction (RT-PCR) was carried out using the following primer sets: the sense primer (5’-TCCTAACAGCTTCCGTTGCT-3’) and antisense primer (5’-ATTCGTCCTCATCCACCTCG-3’) for mouse *Crb2* and the sense primer (5’-GACAACGGCTCCGGCATGTGCA-3’) and the antisense primer (5’-ATGACCTGGCCGTCAGGCAGCT-3’) for mouse *Actb*. One microgram of total RNA was amplified under following conditions: 30 amplification cycles at 98°C for 10 sec, 60°C for 30 sec, and 72°C for 1 min. Amplification was completed with prolonged synthesis at 98°C for 5 min. PCR products were visualized by ethidium bromide staining following electrophoresis on a 1% agarose gel.

### Immunofluorescence and confocal microscopy

For the surface immunostaining of CRB2, 293-CRB2 cells treated with or without tunicamycin were fixed in 4% paraformaldehyde for 15 min and then incubated with blocking buffer (3% bovine serum albumin in PBS). Cells were then incubated with anti-CRB2 antibody (5 μg/ml) recognizing extracellular portion of CRB2 for 60 min at room temperature, followed by incubation with Alexa Fluor 488-conjugated goat anti-rabbit IgG. For the intracellular staining of CRB2, 293-CRB2 cells treated with or without tunicamycin were fixed in 4% paraformaldehyde for 15 min and then incubated with blocking buffer containing 0.5% Tween-20. Cells were incubated with primary antibodies against CRB2 and KDEL at a concentration of 5 μg/ml each for 60 min at room temperature, and then, positive immunostaining was visualized using the secondary antibodies Alexa Fluor 488-conjugated goat anti-rabbit IgG and the Texas Red–X goat anti-mouse IgG, respectively. For the surface immunostaining of MDCK-CRB2 cells, cells were fixed as described above and then incubated with blocking buffer (3% bovine serum albumin in PBS). Cells were reacted with anti-CRB2 antibody as above, followed by a permeabilization procedure using blocking buffer containing 0.5% Tween-20. Cells were then reacted with anti-ZO-1 antibody (5 μg/ml) for 60 min at room temperature. Positive immunostaining was visualized by using the secondary antibodies Alexa Fluor 488-conjugated goat anti-rabbit IgG and the Texas Red–X goat anti-mouse IgG. Paraffin-embedded sections of the neonate and adult kidney were dewaxed, washed with Tris-buffered saline (TBS), and subjected to autoclave heating at 120°C for 10 min in Target Retrieval Solution (Dako) for antigen retrieval. Slides were incubated with blocking buffer (3% bovine serum albumin and 5% goat serum containing 0.5% Tween-20 in TBS) for 60 min and then reacted with the primary antibodies anti-CRB2 (5 μg/ml), anti-phospho-CRB2 (5 μg/ml), anti-CD31 (2 μg/ml), anti-podocalyxin (2 μg/ml) and anti-synaptopodin (1:1) overnight at 4°C. A combination of primary antibodies for double immunofluorescence was performed for CRB2 and synaptopodin, podocalyxin or CD31; phospho-CRB2 and podocalyxin or CD31; podocalyxin and CD31; and podocalyxin and phospho-mTOR. After washing with 0.5% Tween-20 in TBS, the slides were incubated with the appropriate secondary antibodies. The signals were examined under a confocal laser scanning microscope (LSM-510 META, Carl Zeiss Microscopy GmbH, Germany).

### Immunohistochemistry

Kidney cortex samples from newborn mice were fixed in 4% paraformaldehyde for 2 h at 4°C and embedded in paraffin wax. The slides were dewaxed, washed with TBS, and subjected to autoclave heating at 120°C for 10 min in Target Retrieval Solution (Dako) for antigen retrieval. The slides were incubated with 1% H_2_O_2_/TBS for 15 min to quench endogenous peroxidase activity and then treated with blocking buffer (3% bovine serum albumin and 5% goat serum containing 0.5% Tween-20 in TBS) for 60 min. The slides were incubated overnight with an anti-CRB2 antibody (5 μg/ml) or anti-phospho-mTOR antibody (1:100) at 4°C, followed by the detection using an avidin-biotin complex detection system (ABC KIT, Vector Laboratories, Burlingame, CA, USA), and the slides were developed by immersion in 1.4 mmol/L of 3,3’-diaminobenzidine tetrahydrochloride (DAB, Sigma) in TBS.

### Starvation experiment

The 293-wild-type CRB2 cells and 293-phosphomutant-CRB2 cells were seeded in 6-cm poly-d-lysine-coated plates. After 24 h, the cells were rinsed twice with warm PBS. The medium was replaced with serum-free medium, and the cells were incubated for 12 h. The cells were then maintained in Hanks’ balanced salt solution for 2 h, and the medium was then replaced with growth medium for 5 min, 10 min and 15 min. Cell lysates (30 μg protein) were subjected to immunoblotting.

### Immunoblotting

Protein samples from mouse glomeruli and cultured cells were separated by 6% or 12.5% sodium dodecyl sulfate-polyacrylamide gel electrophoresis (SDS-PAGE) and transferred to membranes. After blocking with 3% BSA in TBS, the membranes were incubated with the following primary antibodies at 4°C overnight: anti-CRB2 (0.5 μg/ml), anti-phospho-CRB2 (1.0 μg/ml), anti-phospho-mTOR (1:1000), anti-mTOR (1:1000), anti- RPS6 (0.1 μg/ml), anti-phospho-RPS6 (0.1 μg/ml), anti-Flag (0.1 μg/ml), anti-WT1 (1:200), and anti-β-actin (0.5 μg/ml). The membranes were washed with 0.1% Tween-20 in TBS and subsequently incubated with an HRP-labeled goat anti-rabbit antibody or anti-mouse antibody, and the labeling was detected using a Western Lightning Chemiluminescence reagent kit (Life Science Products, Boston, MA, USA).

### Statistical analysis

All images of immunoblotting were analyzed using ImageJ software. Semi-quantitative analysis of the protein density from the immunoblotting was calculated by the ratio of p-mTOR to mTOR or p-RPS6 to RPS6. The results are shown as the mean ± SE of 3 independent experiments. The statistical significance of the differences was calculated using the ratio paired t-test. Only results with p<0.05 were considered statistically significant.

## Results

### Protein characterization of CRB2 protein in transfected cell lines

**Fig 1:**
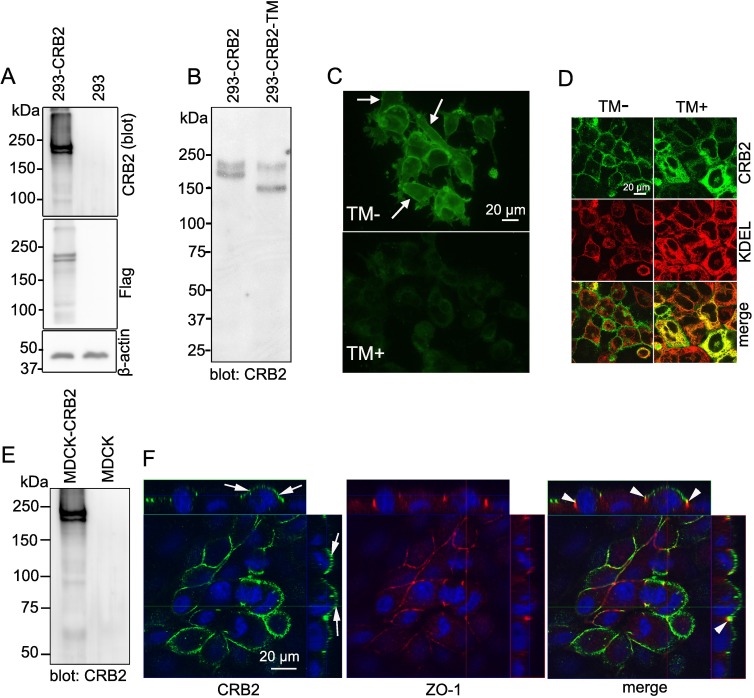
Characterization of mouse CRB2 in cultured cells. A. Immunoblotting of CRB2 in a Flag-tagged-CRB2 expressing 293 cell line (293-CRB2), showing the molecular mass to be approximately 200 kDa as a double band. Immunoblotting of Flag revealed confirmation of the specificity of anti-CRB2 antibody. Immunoblotting of β-actin was performed as the loading control. B. Immunoblotting of CRB2 revealed the reduction in molecular mass by an *N-*glycosylation inhibitor (tunicamycin: TM). C. Immunofluorescence microscopy performed by surface immunostaining and anti-CRB2 antibody recognizing the extracellular portion of CRB2 revealed clear plasma membrane expression of CRB2 (arrow), but not when cells were treated with tunicamycin. D. Intracellular staining followed by mmunofluorescence and confocal microscopy revealed that non-glycosylated CRB2 is retained in the endoplasmic reticulum, colocalizing with KDEL (endoplasmic reticulum marker) in 293-CRB2 cells. E. Immunoblotting of CRB2 in MDCK-CRB2 cells demonstrated almost the same molecular migration for CRB2 as found in 293-CRB2 cells. F. Double immunofluorescence microscopy of CRB2 and ZO-1 using nonpermeabilized MDCK-CRB2 cells was examined and observed by Z stack confocal microscopy. CRB2 was localized to the apical (arrow) and apicolateral (arrowhead) membranes in MDCK-CRB2 cells.

To determine the presence of endogenous CRB2 in cultured podocytes, we first performed RT-PCR of mouse *Crb2* using samples from mouse immortalized podocyte cell lines cultured for 14 days at 37°C. A mouse glomerular sample was used as a positive control and contained *Crb2* transcript, whereas the presence of this transcript in cultured podocytes was not obvious (S1A Fig). Next, immunoblotting of CRB2 was performed to determine the protein expression of CRB2 in this cultured podocyte cell line. WT1 was clearly found in this cell line (arrow), suggesting its reliability for evaluating the CRB2 protein by immunoblotting (S1B Fig). However, the expression of the CRB protein in cultured podocytes was not obvious (S1B Fig). Therefore, we generated a stable cell line constitutively expressing a full-length mouse *Crb2* construct using HEK-293 cells (293-CRB2) and MDCK cells (MDCK-CRB2). Based on immunoblotting using an antibody against the extracellular domain of CRB2, specific immunobands of approximately 200 kDa appeared as a double band when using protein lysates from 293-CRB2 cells but not from 293 cells (Fig 1A). The specificity of these results was confirmed with an anti-FLAG antibody in the presence or absence of FLAG-tagged CRB2 (Fig 1A). Because the predicted molecular mass of the CRB2 protein is approximately 135 kDa, the shift in the electrophoretic migration of CRB2 was most likely due to posttranslational modification. CRB2 is predicted to possess 6 *N*-glycosylation sites (NetNGlyc 1.0: http://www.cbs.dtu.dk/services/NetNGlyc/). When 293-CRB2 cells were treated with the *N*-glycosylation inhibitor tunicamycin, the molecular weight of CRB2 decreased to approximately 140 kDa (Fig 1B). Therefore, the double band was likely due to different *N*-glycosylation patterns. CRB2 is suggested to be a type-1 transmembrane protein [3]. *N*-glycosylation processes play a crucial role in the trafficking of membrane proteins [24]; however, there is no evidence for CRB2 to date. To identify the plasma membrane expression of CRB2, 293-CRB cells were treated with or without tunicamycin, followed by fixation and surface immunostaining using an anti-CRB2 rabbit antibody recognizing the extracellular portion of CRB2. Conventional immunofluorescence microscopy revealed the positive staining of CRB2 on the cell surface in cells treated without tunicamycin (Fig 1C, arrow) but not when cells were treated with tunicamycin. Confocal microscopy following intracellular staining for CRB2 and the endoplasmic reticulum marker KDEL determined that the lack of glycosylation of CRB2 was retained in the endoplasmic reticulum (Fig 1D). Thus, it was concluded that the *N*-glycosylation of CRB2 is crucial for its proper plasma membrane localization. We next examined the protein expression of CRB2 in MDCK cells that are widely used to study the apicobasolateral polarity system [25]. Because MDCK cells do not express endogenous CRB2 protein, we established MDCK-CRB2 cell line. Immunoblotting of CRB2 revealed specific expression as a double band in MDCK-CRB2, and no expression was observed in the control MDCK cells (Fig 1E). To determine the plasma membrane localization of CRB2, MDCK cells were fixed, and nonpermeabilized cells were reacted with primary antibody recognizing the extracellular portion of CRB2, followed by permeabilization and ZO-1 staining. Confocal microscopy showed the localization of CRB2 on the apical (Fig 1F, arrow) and lateral (Fig 1F, arrowhead) sides of the plasma membrane.

### Specific expression of CRB2 in glomerular podocytes

**Fig 2:**
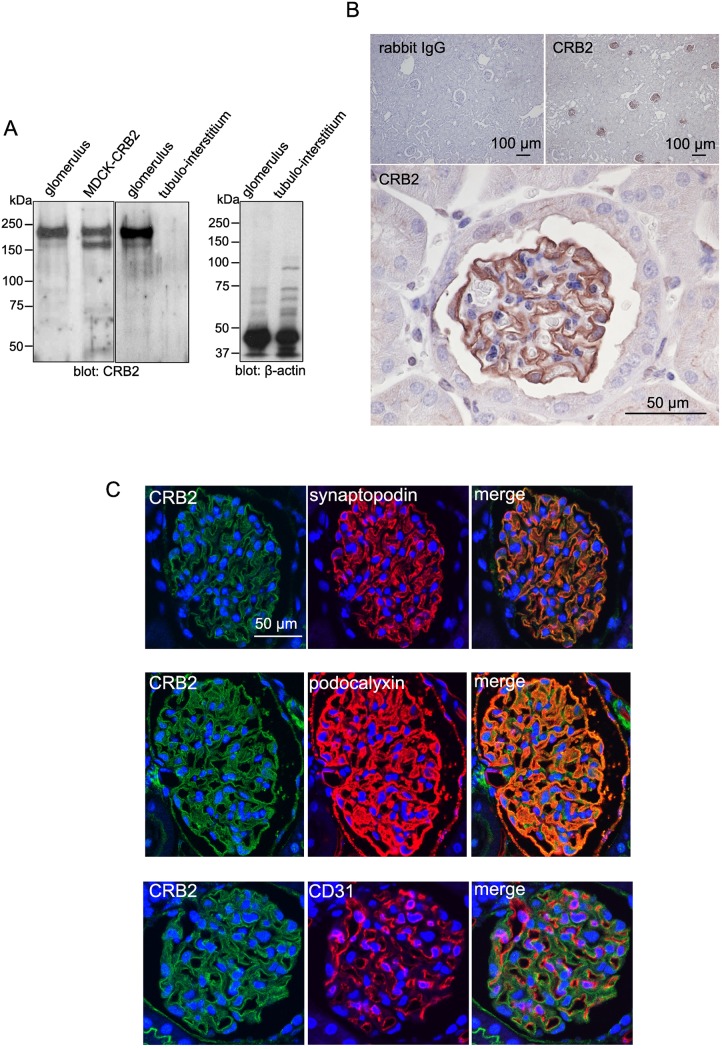
Expression and localization of CRB2 in the adult mouse kidney. A. Immunoblotting in the sample from isolated mouse glomeruli showed specific expression of CRB2 in glomeruli, with the same migration pattern as observed in MDCK-CRB2 cells. Apparent expression of the CRB2 protein was not found in the sample from the tubulointerstitial region. Immunoblotting of β-actin was performed as the loading control. B. Immunohistochemistry of CRB2 in the mouse kidney. Low magnification in the upper panels displayed specific expression of CRB2 in mouse glomeruli (right). Specificity of the antibody reaction was confirmed by the use of rabbit IgG instead of the anti-CRB2 antibody (left). Higher magnification revealed a clear capillary loop pattern of CRB2 in the glomerulus. C. Double immunofluorescence and confocal microscopy revealed overlap of CRB2 with podocyte markers synaptopodin and podocalyxin but not with vascular marker CD31.

To determine CRB2 expression in the adult mouse kidney, we first performed immunoblotting of samples from isolated glomeruli of mice using MDCK-CRB2 cells as a positive control. A double band was obtained using glomerular fractions as well as MDCK-CRB2 cells; however, the upper band was more intense than the lower band in the former samples (Fig 2A). This specific immunoband was not present in the sample from the tubulointerstitial region (Fig 2A), indicating the specific glomerular expression of CRB2 in the adult mouse kidney. Immunohistochemistry revealed the selective expression of CRB2 in the glomeruli in a capillary tuft pattern (Fig 2B). Double immunofluorescence and confocal microscopy displayed complete overlapping of CRB2 with synaptopodin, a podocyte-specific marker in the glomerulus [26]. The colocalization of CRB2 with podocalyxin [27] was also revealed in large part of the glomerulus but not with vascular marker CD31 (Fig 2C). A previous study using antibody produced by glomerular sialoproteins showed that podocalyxin is expressed not only in podocytes but also in glomerular endothelial cells [28]. Compared to this antibody, our anti-podocalyxin antibody was generated by recombinant podocalyxin that does not react with sialoproteins. In order to determine whether the anti-podocalyxin antibody used in the present study reacts with glomerular endothelial cells, we used double immunofluorescence and confocal microscopy for podocalyxin and CD31. As revealed in S2 Fig, the colocalization of podocalyxin with CD31 was determined in only a small part of the glomerulus (arrow), indicating that the antibody used in the present study reacts mainly with podocalyxin of podocytes. Taken together, we concluded that CRB2 is specifically expressed in the podocyte of the mature glomerulus.

### Migration of CRB2 in the plasma membrane in developing podocytes

**Fig 3:**
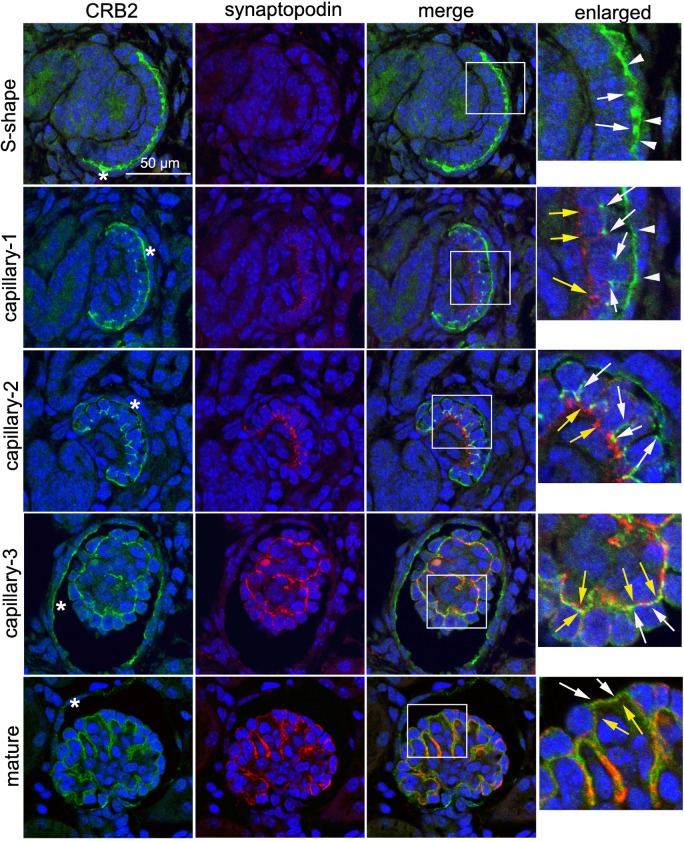
Dynamic migration of CRB2 in developing podocytes. Double immunofluorescence for CRB2 and synaptopodin followed by confocal microscopy in the newborn mouse kidney. CRB2 was located both in the glomerular visceral (S-shape, white arrow) and parietal (S-shape, arrowhead) immature epithelium, where immunostaining was observed at the apicolateral and apical membranes, respectively. At the early capillary stage, CRB2 was predominantly localized at apical (capillary-1, arrowhead) and apicolateral (capillary-1, white arrow) regions, whereas synaptopodin was found at the basolateral side in premature podocytes (capillary-1, yellow arrow). Plasma membrane localization of CRB2 on parietal epithelium was preserved (capillary-1, arrowhead). In more mature podocytes (capillary-2, capillary-3, and mature), CRB2 had moved down to the basolateral side and eventually colocalized with synaptopodin at the foot processes (CRB2, white arrow; synaptopodin, yellow arrow). Through the capillary stage, CRB2 expression in glomerular parietal cells was positive, though its expression became faint in the mature glomerulus (asterisk in CRB2 lane). An enlarged view is displayed within the rectangle.

**Fig 4:**
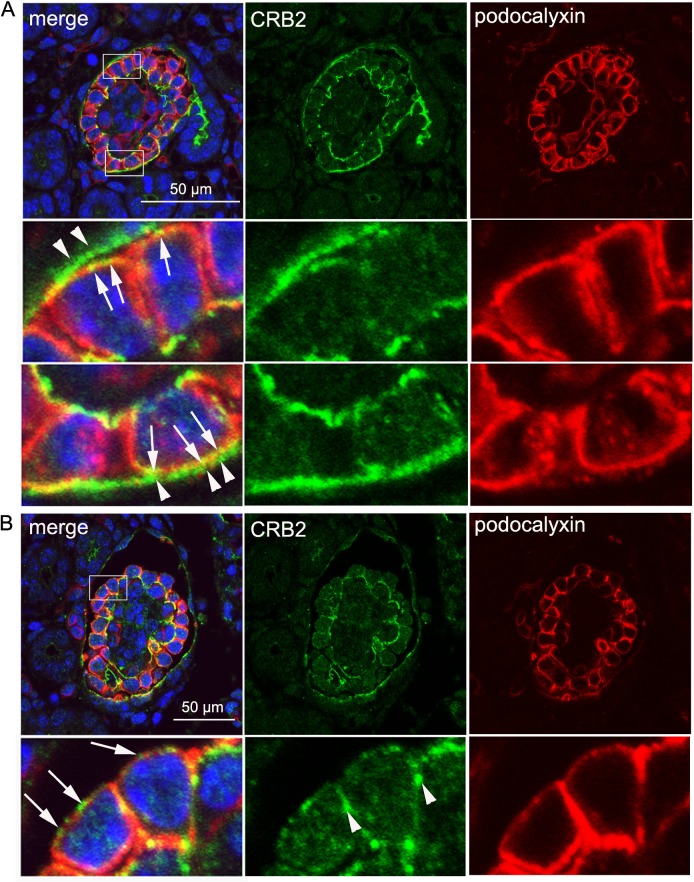
CRB2 is colocalized with podocalyxin in immature glomeruli. Double immunofluorescence microscopy for CRB2 and podocalyxin using the newborn mouse glomerulus was performed followed by confocal microscopy. A. Clear localization of CRB2 in the plasma membrane of parietal epithelial cells (arrowhead) and the apical membrane of podocytes (arrow) was shown. B. CRB2 was localized both to the apical (arrow) and lateral (arrowhead) sides of the plasma membrane of immature podocytes. An enlarged view is displayed within the rectangle.

Confocal microscopy following double immunofluorescence staining of CRB2 and synaptopodin was performed to determine the expression pattern of CRB2 in the developing glomerulus. Synaptopodin is not expressed during the S-shaped body stage [26]. At this stage, CRB2 was located in both the glomerular visceral (Fig 3, S-shape, white arrow) and parietal (Fig 3, S-shape, arrowhead) epithelium, where immunostaining was observed at the apicolateral and apical membranes, respectively. At the early capillary stage, CRB2 predominantly localized at the apicolateral region (Fig 3, capillary-1, white arrow) of premature podocytes, whereas synaptopodin was expressed on the basolateral side (Fig 3, capillary-1, yellow arrow). Plasma membrane localization of CRB2 on the parietal epithelium was preserved (Fig 3, capillary-1, arrowhead). In more mature podocytes, CRB2 moved down to the basolateral side and eventually colocalized with synaptopodin at foot processes (Fig 3, capillary-2, -3 and mature, CRB2: white arrow, synaptopodin: yellow arrow). Through the capillary stage, CRB2 expression in the glomerular parietal cell (Bowman’s cell) was intense, although it became faint in the mature glomerulus (Fig 3, asterisk in CRB2 lane). To more precisely determine the localization of CRB2 in the premature podocyte, newborn kidney sections were double-immunostained for CRB2 and podocalyxin. Confocal microscopy showed clear localization of CRB2 at the plasma membrane of parietal epithelial cells (Fig 4A, arrowhead) and the apical membrane of podocytes (Fig 4A, arrow). Again, CRB2 clearly localized both at the apical side (Fig 4B, arrow) and the lateral side (Fig 4B, arrowhead) of the plasma membrane of podocytes.

### Identification of tyrosine phosphorylation of CRB2 in cultured cells and developing podocytes

**Fig 5:**
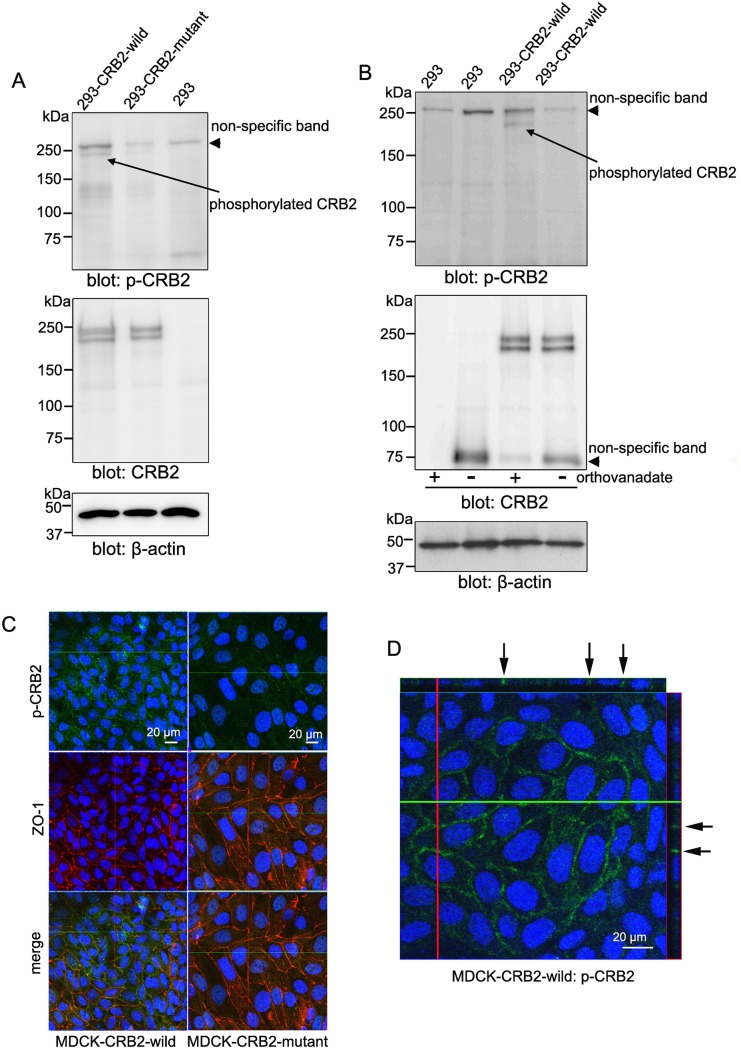
Characterization of the anti-tyrosine phosphorylated CRB2 antibody and CRB2 mutant cells lacking tyrosine phosphorylation. A. Immunoblotting using the anti-phospho-CRB2 antibody revealed a positive immunoband (arrow) from 293-CRB2-wild cells but a very faint immunoband from 293-CRB2-phospho-mutant cells (293-CRB-mutant). The immunoband migrating at 250 kDa was determined to be a non-specific band because this band was also observed in 293 cells (arrowhead). B. The absence of a tyrosine-phosphatase inhibitor (orthovanadate) led to loss of CRB2 tyrosine phosphorylation in samples from wild-CRB2 cells (arrow). C and D. MDCK-CRB2 cells were subjected to double immunofluorescence for phospho-CRB2 and ZO-1. Confocal microscopy showed specific localization of tyrosine-phosphorylated CRB2 on the lateral side of the plasma membrane in MDCK-CRB2-wild cells (D, arrow) but not in MDCK-CRB2-phospho-mutant cells (MDCK-CRB2-mutant).

**Fig 6:**
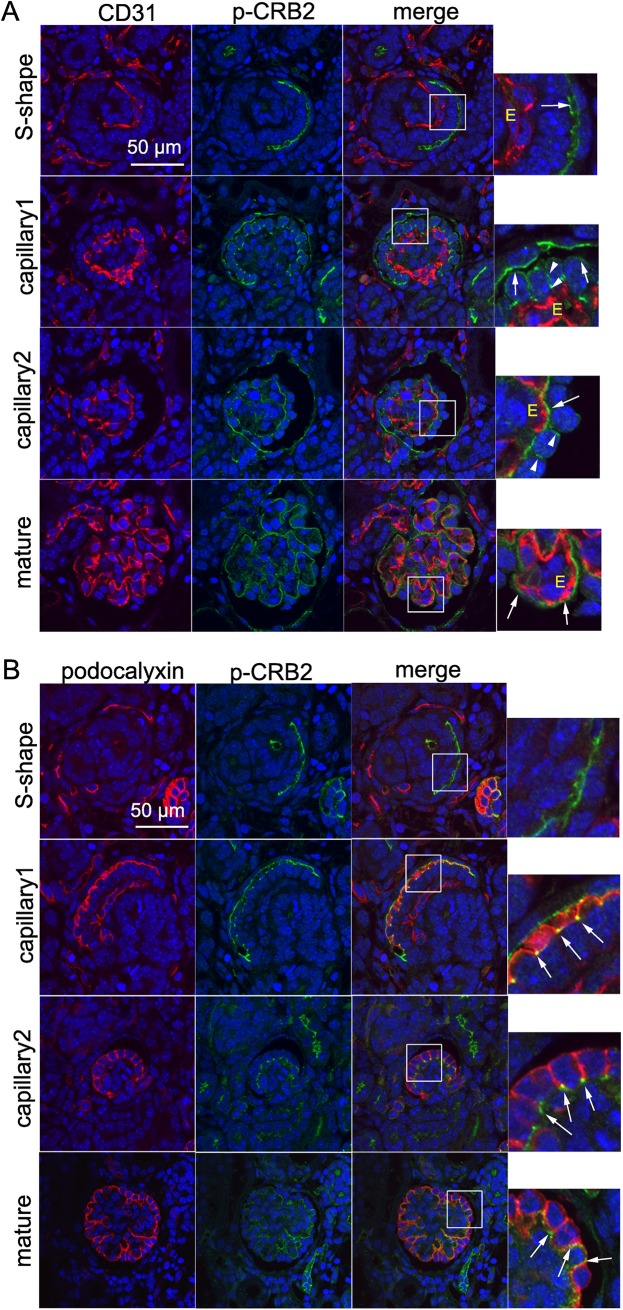
Dynamic migration of CRB2 tyrosine phosphorylation in developing podocytes. A. Double immunofluorescence and confocal microscopy for tyrosine-phosphorylated CRB2 and CD31 was performed. Clear positive staining of tyrosine-phosphorylated CRB2 in podocyte precursor cells in the S-shaped body (arrow) is shown, which became intense in the basolateral membrane at the capillary stage (capillary 1, arrowhead). The clear capillary tuft pattern of tyrosine-phosphorylated CRB2 is present in the mature glomerulus (capillary 2 and mature, arrow). E, endothelial cell. An enlarged view is displayed within the rectangle. B. Double immunofluorescence and confocal microscopy for tyrosine-phosphorylated CRB2 and podocalyxin was performed. Partial overlapping of tyrosine-phosphorylated CRB2 and podocalyxin signals is shown from the apical side to the basolateral side during podocyte development (arrow). In the mature glomerulus, CRB2 tyrosine phosphorylation was revealed as a capillary tuft pattern, likely in foot processes (mature, arrow). An enlarged view is displayed within the rectangle.

Tyrosine phosphorylation is a fundamentally important modification in eukaryotic cells that controls many cellular processes including development and differentiation [29]. We sought to determine the involvement of tyrosine-phosphorylated CRB2 in developing podocytes. To address this aim, we generated a specific antibody against tyrosine-phosphorylated CRB2 (phospho-CRB2) as well as 293-CRB2 cells expressing CRB2 lacking the tyrosine phosphorylation site (293-CRB2-mutant). As shown in Fig 5A, the immunoblotting of the anti-phospho-CRB2 antibody identified an immunoband in 293-CRB2-wild-type cells but a very weak band in 293-CRB2-mutant cells (arrow). Moreover, the immunobands were not visible when protein samples from 293-CRB2-wild-type cells were subjected to immunoblotting in the absence of a tyrosine-phosphatase inhibitor (sodium orthovanadate), indicating the specificity of the anti-phospho-CRB2 antibody (Fig 5B). To determine the subcellular localization of phosphorylated CRB2, MDCK-CRB2 cells were subjected to double immunofluorescence for phosphorylated CRB2 and ZO-1. Confocal microscopy revealed the specific localization of tyrosine-phosphorylated CRB2 on the lateral side of the plasma membrane in MDCK-CRB2-wild-type cells but not in MDCK-CRB2-mutant cells (Fig 5C and 5D, arrow). To identify the expression pattern of phosphorylated CRB2 in the mouse neonatal kidney, double immunofluorescence with phospho-CRB2 and the endothelial marker CD31 was performed followed by confocal microscopy. A weak but clear signal of tyrosine phosphorylated CRB2 was observed on the apical side of podocyte precursor cells in the S-shaped body (Fig 6A, S-shape, arrow). At the early capillary stage, the site of tyrosine phosphorylated CRB2 moved down to the basolateral site (Fig 6A, capillary 1, arrowhead), and its intensity on the apical side was reduced (Fig 6A, capillary 1, arrow). In the more developed capillary stage, tyrosine phosphorylated CRB2 was maintained on the lateral side of podocyte body (Fig 6A, capillary 2, arrowhead), whereas intense expression was found in the foot processes in a capillary tuft pattern (Fig 6A, capillary 2, arrow). Finally, mature-stage glomeruli revealed a clear foot process pattern of tyrosine phosphorylated CRB2 (Fig 6A, mature, arrow). To further confirm the subcellular localization of tyrosine phosphorylated CRB2 in developing podocytes, double immunofluorescence for phospho-CRB2 and podocalyxin was performed. Similar to the results presented in Figs 3 and 4 showing the CRB2 extracellular domain, confocal microscopy indicated clear overlapping of tyrosine-phosphorylated CRB2 with podocalyxin from the apical side to the basolateral side in premature podocytes (Fig 6B, capillary 1 and 2, arrow). In the mature glomerulus, tyrosine phosphorylated CRB2 appeared as a capillary tuft pattern, likely in foot processes (Fig 6B, mature, arrow). These results indicate that tyrosine phosphorylation dynamically occurs in the intracellular region of CRB2 in podocytes throughout their developmental stages.

### Association of CRB2 tyrosine phosphorylation with mTORC1 activation in developing podocytes

**Fig 7:**
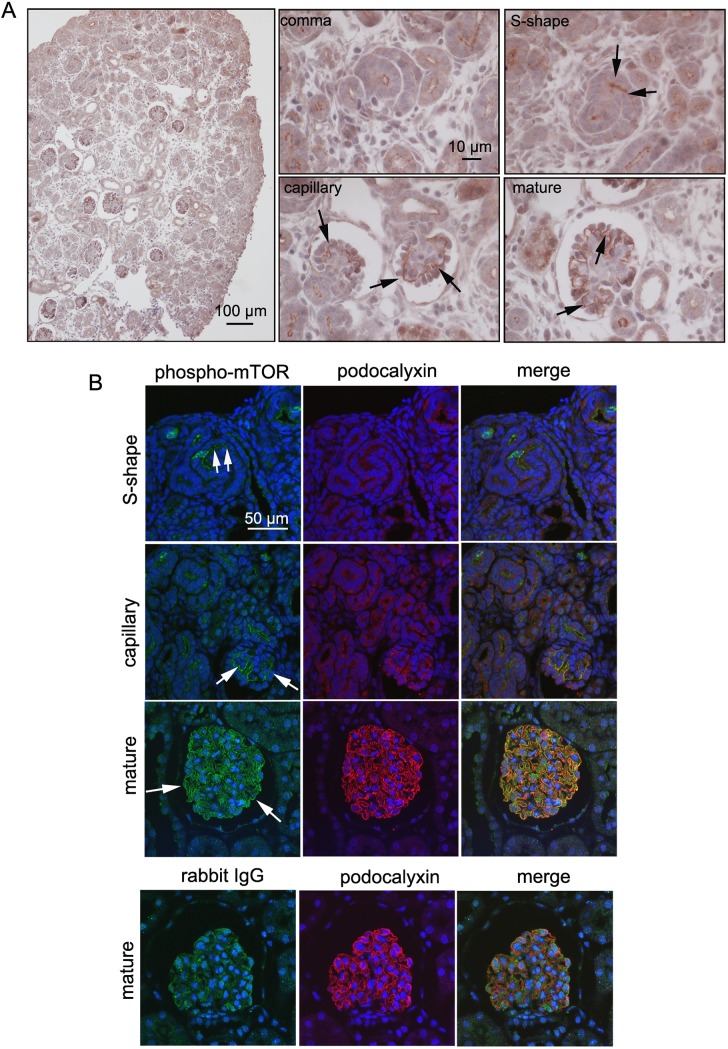
Expression of mTOR phosphorylation in developing podocytes. A. Immunohistochemistry demonstrated predominant expression of phosphorylated mTOR, commencing at the S-shaped body stage in podocytes of the newborn mouse kidney (arrow). In capillary and mature stages, mTOR phosphorylation occurred in podocytes, including in foot processes. B. Double immunofluorescence and confocal microscopy for phosphorylated mTOR and podocalyxin. Colocalization of phosphorylated mTOR with podocalyxin was seen in the capillary and mature stages. Arrows indicate positive staining of phosphorylated mTOR in immature and mature podocytes. Use of rabbit IgG instead of the primary antibody (phospho-mTOR) resulted in no apparent positive staining. An enlarged view is displayed within the rectangle.

A previous report indicated that phosphorylated mTOR (p-mTOR) is present in the mature glomerular podocytes of adult mice [30]. To more precisely determine the expression patterns of p-mTOR in developing podocytes, the newborn mouse kidney was subjected to immunohistochemistry. As shown in Fig 7A, the intensity of p-mTOR in podocyte precursor cells was faint in the comma-shaped body and slightly increased in the S-shaped body (Fig 7A, arrow). In the capillary and mature stages, the activation of p-mTOR became more intense and was revealed as a capillary tuft pattern (Fig 7A, arrow). Colocalization of p-mTOR with podocalyxin was further confirmed by double immunofluorescence staining and confocal microscopy, indicating mTOR phosphorylation in mature podocytes (Fig 7B, arrow). Rabbit IgG used as a negative control did not result in any specific pattern, indicating the specificity of p-mTOR staining (Fig 7B).

**Fig 8:**
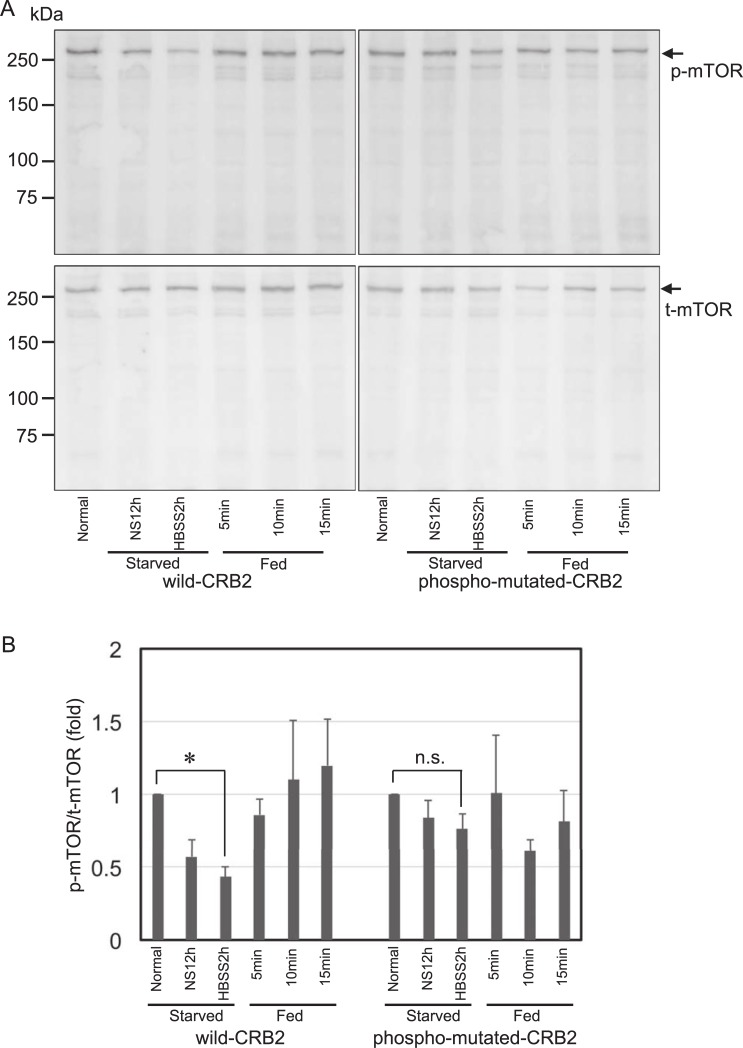
Lack of CRB2 tyrosine phosphorylation leads to the reduced sensitivity of mTOR activation in response to energy starvation. A. Cells were subjected to an energy starvation experiment. Representative immunoblotting of phospho- and total mTOR (p-mTOR and t-mTOR, respectively) was shown. Protein samples from wild-CRB2 and phospho-mutated-CRB2 cells (30 μg each) were separated. B. Semi-quantitative analysis of the protein density from the immunoblotting in A was calculated by the ratio of p-mTOR to t-mTOR. The results are shown as the mean ± SE of 3 independent experiments. NS: non-serum, HBSS: Hanks' balanced salt solution. * *P*<0.05. n.s. indicates not significant.

**Fig 9:**
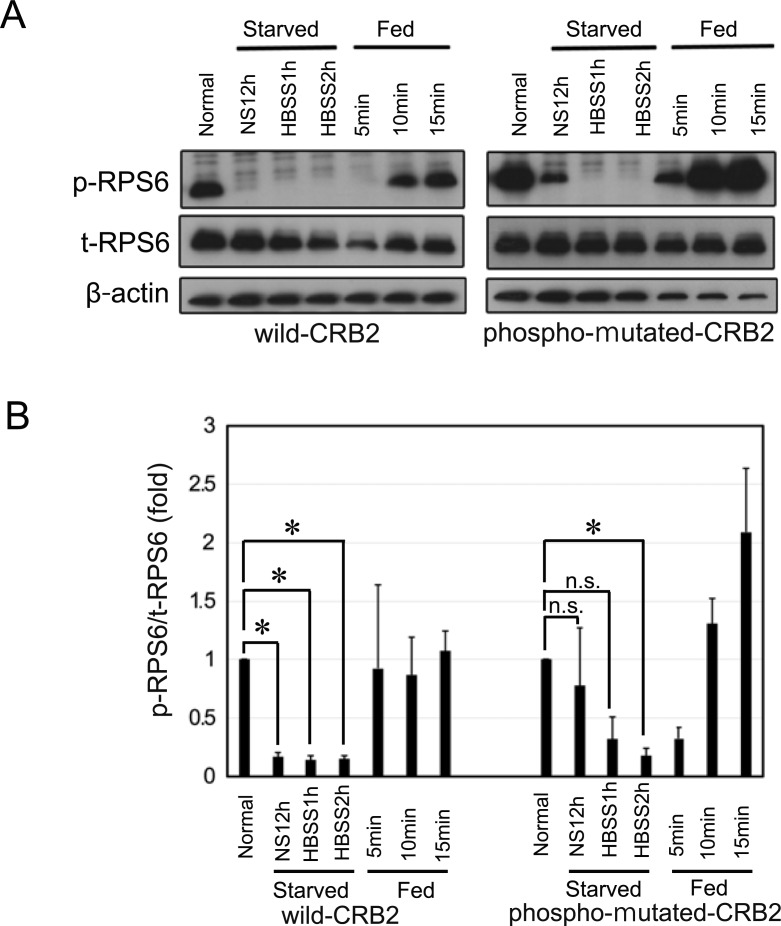
Lack of CRB2 tyrosine phosphorylation leads to the reduced sensitivity of S6-ribosomal protein (RPS6) activation in response to energy starvation. Representative immunoblotting with phospho- and total RPS6 (p-RPS6 and t-RPS6, respectively), a downstream effector of mTORC1 activation, was shown. Protein samples from wild-type CRB2 and phospho-mutated-CRB2 cells (30 μg each) were loaded. B. Semi-quantitative analysis of the protein density from immunoblotting in A was calculated by the ratio of p-RPS6 to t-RPS6. The results are shown as the mean ± SE of 3 independent experiments. NS: non serum, HBSS: Hanks' Balanced Salt Solution. * *P*<0.05. n.s. indicates not significant.

As CRB3 reportedly represses the mTOR pathway [16], we sought to determine whether CRB2 is also involved in the mTOR pathway. We employed a starvation and nutrient restoration assay using 293-CRB2 cells to determine whether tyrosine phosphorylation of CRB2 is associated with mTOR activation. Equal volumes of protein samples were separated and immunoblotted, followed by analysis of the ratio of phospho-mTOR (p-mTOR) to total-mTOR (t-mTOR). Immunoblotting revealed that non-serum medium (NS) treatment followed by Hanks' balanced salt solution (HBSS) incubation reduced the ratio of p-mTOR to t-mTOR, whereas growth medium restoration rapidly restored the ratio, indicating the reliability of these experimental procedures (Fig 8A and 8B). When the ratio of p-mTOR to t-mTOR under normal conditions (Normal) was set to 1.0, the ratios of the samples from the wild-CRB2 cells starved in NS for 12 h (NS12h) and Hanks' balanced salt solution for 2 h (HBSS2h) were 0.57 ± 0.11 (mean ± SE) and 0.43 ± 0.07, respectively (Fig 8A and 8B). In this wild-CRB2 cell, there was a significant reduction in mTOR phosphorylation of NS12h and HBSS2h when compared to Normal (*P*<0.05). By contrast, the samples from the phospho-mutated-CRB2 starved cells in NS12h and HBSS2h resulted in values of 0.84 ± 0.12 and 0.77 ± 0.10, respectively, and there was no significant difference in NS12h and HBSS2h when compared to Normal (Fig 8A and 8B). Moreover, during growth medium restoration, wild-CRB2 cells exhibited time-dependent recovery of mTOR activation as follows: 5 min, 0.86 ± 0.10; 10 min, 1.10 ± 0.40; and 15 min, 1.20 ± 0.32 (Fig 8A and 8B). In contrast, phospho-mutated-CRB2 cells were not similar to wild-CRB2 cells: 5 min, 1.0 ± 0.4; 10 min, 0.61 ± 0.08; and 15 min, 0.81 ± 0.21 (Fig 8A and 8B). A ratio of phospho-RPS6 (p-RPS6) to total-RPS6 (t-RPS6), a downstream effector of mTORC1 activation, was also examined. Representative of immunoblotting for p-RPS6 and t-RPS6 was shown in Fig 9A and their whole blots were revealed in S3 Fig. The ratio of p-RPS6 to t-RPS6 under normal conditions (Normal) was also set to 1.0. Clearly, in wild-CRB2 cells, there was a significant reduction in RPS6 phosphorylation of all starved samples (NS12h, HBSS1h, and HBSS2h) when compared to Normal (*P*<0.05). On the other hand, a significant reduction of RPS6 phosphorylation in phospho-mutated-CRB2 cells was seen only in the samples from HBSS2h when compared to Normal (Fig 9B), Taken together, the data indicated that a lack of tyrosine phosphorylation of CRB2 leads to the reduced sensitivity of the mTORC1 pathway in response to energy starvation.

## Discussion

Our goal in this study was to uncover the molecular basis of the involvement of CRB2 in the cellular processes of podocyte differentiation. Our first novel finding was that the plasma membrane assembly of CRB2 requires *N*-glycosylation, similar to nephrin, a major component of the SD [31]. Although CRB3 is also an *N*-glycosylated protein, its posttranslational modification is not essential for plasma membrane localization [8]. In addition to its crucial role in protein trafficking, the biological role and the ligands of sugars on the extracellular domain of CRB2 in podocyte precursor cells and mature podocytes remain largely elusive.

The second novel finding was the protein expression pattern of CRB2 in developing glomeruli, which started at podocyte precursor cells and continued in mature podocytes. The overall expression patterns for CRB2 in developing glomeruli were quite similar to those of Scribble, a cytoplasmic scaffold protein that is a component of another polarity complex and exhibits movement downward from the lateral side of immature podocytes to the basolateral side of the foot processes of mature podocytes [32,33]. Nonetheless, the mechanism for the translocation of CRB2 during podocyte differentiation remains largely obscure. It is known that the highly conserved cytoplasmic tails of CRB proteins play a crucial role in cellular functions, including cytoskeleton reorganization. CRB proteins form a protein complex with PALS1 and PATJ and with moesin by binding to their FERM motif [34]. Moesin is a member of the ERM family, which includes ezrin and radixin and most likely functions as a cross-linker between the plasma membrane and actin-based cytoskeleton [35]. Moesin is also known to be expressed in the slit diaphragm-enriched fraction of the mouse glomerulus [36]. It is well-recognized that actin reorganization is a key process in podocyte differentiation [22, 37]. Although our data did not reveal direct evidence of cross-talk between CRB2 and the cytoskeleton network, it is possible that CRB2 may be involved in cytoskeleton organization by interacting with the ERM family in developing podocytes.

Our third novel finding was the role of CRB2 tyrosine phosphorylation in developing podocytes. Interestingly, we found a different response of mTOR activation against energy stress between wild-CRB2 cells and tyrosine phospho-mutated-CRB2 cells. The starvation experiment indicated that the absence of tyrosine phosphorylation of CRB2 led to the reduced sensitivity of mTOR activation to energy depletion compared to its presence. Moreover, energy restoration did not completely restore mTOR activation in phospho-mutated CRB2 cells even at 15 min after growth medium supplementation. In addition, a lack of tyrosine phosphorylation of CRB2 led to a similar response of RPS6 activation, a downstream molecule of mTORC1, to that of mTOR. Previous studies indicated that CRB3 interacts with PATJ and tuberous sclerosis complex protein 1 or 2, central players in the mTORC1 pathway [16]. In addition, mTORC1 activity increases when PATJ is deleted in CRB3-expressing cells [16]. mTOR is an evolutionarily conserved serine/threonine kinase, the activation of which stimulates both the initiation and elongation phases of mRNA translation and increases ribosome biogenesis, thereby accelerating cell size enlargement [38]. Our immunofluorescence analyses indicated that the timing and location of CRB2 tyrosine phosphorylation were similar to those of mTOR activation in developing podocytes. The biological role of mTORC1 activation in developing podocytes remains largely obscure. There is also evidence that excess mTORC1 activation is possibly involved in cellular dysfunction, which leads to congenital and acquired diseases, including those affecting the kidney [39, 40]. The current study used cell lines from humans and canines, which were transfected with a mouse CRB2 construct. We have not determined how the CRB2-mTROC1 axis is conserved among these species, particularly in podocytes. Therefore, several limitations of this study do not lead to the certainty of the conclusion that CRB2 functionally interacts with the mTORC1 pathway in podocytes. Nonetheless, the results from the present study revealed the possible association of CRB2 tyrosine phosphorylation with the mTORC1 pathway in developing podocytes. We propose that CRB2 may act as an upstream regulator of mTORC1 activation in developing podocytes. Therefore, the upstream molecules and signaling pathways that regulate CRB2 phosphorylation should be clarified to further understand CRB2 function in podocyte biology.

## Supporting information

S1 Fig Mouse podocyte cell line does not express endogenous CRB2. A. RT-PCR of mouse CRB2 using samples from a mouse immortalized podocyte cell line and isolated mouse glomeruli as a positive control. The 530 bp product indicates the CRB2 transcript. β-actin is shown as a loading control. B. Immunoblotting of WT1, CRB2 and β-actin. The immortalized podocyte cell line expresses WT1, a podocyte marker, migrating at approximately 50 kDa (arrow). No obvious endogenous expression of CRB2 protein is seen. β-actin is revealed as a loading control.
(EPS)

S2 Fig Double immunofluorescence and confocal microscopy of podocalyxin and CD31. Colocalization of podocalyxin with CD31 is only observed in a small part of the glomerulus (arrow), indicating that the anti-podocalyxin antibody used in the present study mainly reacts with the podocyte. An enlarged view is displayed within the rectangle.
(EPS)

S3 Fig Whole blots of Fig 9A. (EPS)
